# Extraction, Purification, Structural Characteristics, Biological Activities and Pharmacological Applications of Acemannan, a Polysaccharide from *Aloe vera*: A Review

**DOI:** 10.3390/molecules24081554

**Published:** 2019-04-19

**Authors:** Chang Liu, Yan Cui, Fuwei Pi, Yuliang Cheng, Yahui Guo, He Qian

**Affiliations:** 1 State Key Laboratory of Food Science and Technology, School of Food Science and Technology, Jiangnan University, Wuxi 214122, China; liuchang670250789@gmail.com (C.L.); pifuwei@jiangnan.edu.cn (F.P.); wxfoodcyl@126.com (Y.C.); 2 Synergetic Innovation Center for Food Safety and Nutrition, Jiangnan University, Wuxi 214122, China; 3 Institute of Agricultural Products Processing, Key Laboratory of Preservation Engineering of Agricultural Products, Ningbo Academy of Agricultural Sciences, Ningbo 315040, China; cuiyan1605@126.com

**Keywords:** *Aloe vera*, polysaccharide, acemannan, biological activity, applications, structure

## Abstract

*Aloe vera* is a medicinal plant species of the genus Aloe with a long history of usage around the world. Acemannan, considered one of the main bioactive polysaccharides of *Aloe vera*, possesses immunoregulation, anti-cancer, anti-oxidation, wound healing and bone proliferation promotion, neuroprotection, and intestinal health promotion activities, among others. In this review, recent advancements in the extraction, purification, structural characteristics and biological activities of acemannan from *Aloe vera* were summarized. Among these advancements, the structural characteristics of purified polysaccharides were reviewed in detail. Meanwhile, the biological activities of acemannan from *Aloe vera* determined by in vivo, in vitro and clinical experiments are summarized, and possible mechanisms of these bioactivities were discussed. Moreover, the latest research progress on the use of acemannan in dentistry and wound healing was also summarized in details. The structure-activity relationships of acemannan and its medical applications were discussed. Finally, new perspectives for future research work on acemannan were proposed. In conclusion, this review summarizes the extraction, purification, structural characteristics, biological activities and pharmacological applications of acemannan, and provides information for the industrial production and possible applications in dentistry and wound healing in the future.

## 1. Introduction

Polysaccharides are a kind of carbohydrate with high molecular weight, which represent a major class of bioactive molecules derived from microorganisms, animals, or plants [1]. Polysaccharides are extensively used in various healthcare products and medicines because of their notable and excellent bioactivities, such as antimicrobial [2], antitumor [3], antiviral [4], and antioxidant activities [5]. In addition, polysaccharides are among the natural biopolymers found on Earth [6], which are widely used as biomaterials for wound healing [7], drug delivery [8] and tissue engineering [9]. *Aloe vera* is one of the few natural plants that are very abundant in polysaccharides [10].

*Aloe vera* is one of the most popular medicinal plants, widely used for the prevention or treatment of skin diseases, metabolic diseases, cardiovascular diseases and cancers throughout the world. Numerous studies have reported that aloe leaf possesses numerous functions, which are attributed to the presence of polysaccharides, such as immunomodulation, antimicrobial, antiviral, anti-cancer, anti-inflammatory properties [11,12,13]. Polysaccharide acetylation can lead to many changes in the function of polysaccharides [14]. Acemannan, a β-(1,4)-acetylated soluble polymannose, is the major bioactive polysaccharide of *Aloe vera,* from which gel and skin it is extracted [15]. In the past few decades, acemannan has been reported to have many pharmacological and biological applications in medical and industrial fields, such as oral diseases [16], metabolic diseases, cardiovascular diseases, tumor diseases [17]. Recently, research on acemannan has focused on dentistry and wound healing. Acemannan has been used for the treatment of wounds and alveolar osteitis using protocols approved by the US Food and Drug Administration (FDA) [7,18]. More importantly, more and more studies are paying attention to the applications of acemannan in new materials and drug delivery [19]. 

On the one hand, although acemannan has been extensively investigated, there is the only one mini review mainly concentrated on the pharmacological advancement of acemannan from *Aloe vera* [15] and no comprehensive review of the extraction, purification, biological activities of acemannan. Thus, summarizing the information on acemannan in the extraction, purification, biological activities is of great value.

On the other hand, while the latest research on acemannan was focused on dentistry and wound healing, the data concerning dentistry and orthopedics is scattered. In view of this, summary of the role and mechanism of acemannan in the latest medical research is necessary. Meanwhile, the relationships between the activities of acemannan and its structure have not been completely elucidated, which limits its application. Thus, summarizing the different functions and mechanisms of acemannan in medical research is of great value.

Hence, the aim of the present review is to summarize the recent advances in extraction, purification, structural characteristics and pharmacological activities of acemannan, respectively, discuss the relationship between the activity and structure, and summarize the applications in terms of materials and medicines.

## 2. Extraction, Separation and Purification of Acemannan

### 2.1. Factors Affecting Acemannan Production and Structure

Gel and skin of aloe are the main sources of acemannan, which has β-1,4 linkages and a variable degree of acylation [20,21]. Polysaccharides constitute most of the dry matter of aloe. Acemannan is a kind of storage polysaccharide, an acetylated glucomannan, which is located in the protoplasts of parenchyma cells that contain many polysaccharides in the cell wall matrix. Carbohydrate analysis of water soluble residues in aloe leaves showed that glucomannan was mainly located in the cell walls of aloe leaves [22,23].

Aloe acemannan variability depends greatly on the species and cultivation conditions. The content of the β-polysaccharide fraction, however, was significantly higher in *A. vera* than *A. arbor*escens [24]. Meanwhile, the content of acemannan and the degree of acetylation also depend on different planting conditions. The irrigation of the plant affects the amount of polysaccharides. The mannose decreases by 41% and acemannan does not undergo deacetylation due to a water deficit of up to 60% [25]. When the aloe is irrigated with seawater, 42% seawater stress treatment only reduces the polysaccharide concentration in the base leaves, without lowering the polysaccharide concentration in the upper and middle parts [26]. Moreover, the growth age and harvest season of aloe also affect the content of polysaccharide. Acemannan is more abundant in three years old *Aloe vera* plants than in four years old and two years old ones, and growing under increasing light intensities results in higher acemannan concentrations in *A. vera* and *A. arborescens* according to semi-quantitative estimation and ^1^H-NMR spectroscopy analysis [24,27].

The potential use of acemannan often involves some type of processing, which can include heating, drying, pasteurization and dehydration [10]. The main effects of spray drying, industrial freeze-drying, refractive window drying and radiation zone drying on the main bioactive polysaccharide acemannan in aloe gel were studied. All the drying processes significantly reduced the yield of acemannan (∼40%). Methylation analysis showed that mannose deacetylation (>60%) was confirmed by ^1^H-NMR analysis [28]. The effects of heat treatment and dehydration on the physicochemical properties of bioactive acemannan polysaccharide in *Aloe barbadensis* Miller parenchyma at different temperatures (30–80 °C) were studied and indicated that the modification of acemannan is especially significant at 60 °C. A new food drying method, far infrared radiation (FIR) and high-voltage electric field (HVEF), was developed to dry aloe in hot air. The study found that the polysaccharide remained fairly stable up to around 70 °C. At a certain temperature, the higher the electric field intensity, the higher the content of acemannan in the sample, which may be related to the shorter drying time. The content of mannose increased with the increase of electric field intensity and temperature [29]. Heating mainly increased the average molecular weight of polysaccharides from 45 kDa to 81 kDa, which may be mainly due to structural modifications, such as deacetylation and losses of galactose-rich side-chains from the mannose backbones detected through methylation analysis [23]. There are also related studies that show that the losses of galactosyl residues and deacetylation process, would result in mannose-rich chains of higher molecular weight (MW). Distribution of the acetyl groups and galactosyl units in the main chain can have a significant effect on the physical and biological characteristics of the acemannan [30]. In conditions of 65, 75 and 85 °C, a pasteurisation process of 15 min and 25 min was carried out on acemannan, to promote its physical and chemical modification, respectively. The pasteurization method can improve the yield of acemannan [30]. 

### 2.2. Exaction of Acemannan

**Figure 1:**
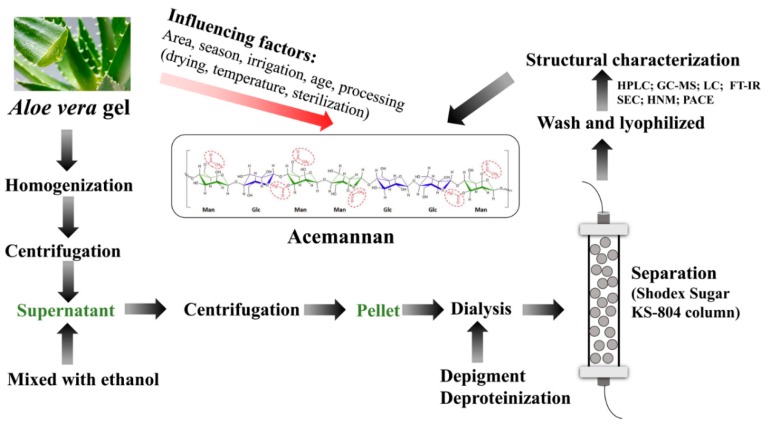
Schematic representation of extraction, separation purification and structure characterization of acemannan from *Aloe vera*.

Acemannan, found in internal leaf aloe gel, is a polysaccharide composed of β-(1,4)-linked highly acetylated mannoses, β-(1,4)-linked glucose and α-(1,6)-linked galactose [21,31]. Since the bioactive components of aloe including acemannan always have considerably different exaction methods (Table 1). The separation process is shown in Figure 1, where the molecular structure is taken from [32].

Generally, extraction in hot water and ethanol is the classic, most convenient method of laboratory extraction, and has been widely used in industry [29,33]. Briefly, the water exaction method includes cleaning, homogenization, separation, and centrifugation of *Aloe vera*. The supernatant was collected and mixed with absolute ethanol at a ratio of 1:3 for 12 h. The white precipitate was collected and centrifuged. Acemannan was collected by dialysis and freeze-drying as white opaque particles [34]. The ratio of liquid to solid has an important effect on the yield of conventional water extraction. The range of extraction temperature with time is usually in the range of 80–100 °C, 0.5–6 h [35,36]. However, water extraction, the most popular approach, always has disadvantages of long times and high temperatures, low efficiency, and possible polysaccharides degradation, resulting in large consumptions of energy and time. Therefore, it is necessary to improve the extraction conditions. Numerous studies have proved aloe polysaccharide was partially digested with cellulose [37].

### 2.3. Separation and Purification of Acemannan

After extraction, the crude extracts are mainly obtained by ethanol precipitation (Table 1). As such polysaccharides usually contain proteins, pigments and small molecular substances, further separation and purify is necessary to get acemannan. Separation and purification of crude extracts by anion exchange chromatography coupled with gel permeation chromatography (DEAE-Sephadex A-25 column) [38,39,40] and normal membrane separation were done [41]. However, considering the long fractionation time, low cost, membrane vulnerability, gradient ethanol precipitation method and gradient ammonium sulfate precipitation method were adopted in recent years. Besides, the membranes with special structure and function are also used in the separation and purification of acemannan. Acemannan was fractionated with ultrafiltration cells from an Amicon with the corresponding molecular weight cut-off membrane according to the molecular size [14,42]. Moreover, electrospun cellulose acetate membrane was used as an alternative carrier for separation of crude extracts by electrophoresis. By controlling the porosity and pore size of membrane, it was used as a simple technical advantage for separation of high molecular weight and near molecular weight polysaccharides [43].

### 2.4. Structural Characterization Method and Characteristics of Acemannan

Acemannan was determined by high performance liquid chromatography (HPLC, BIOSEP SECH400 column, Table 1). Then the structure of acemannan was analyzed by ultraviolet (UV) spectroscopy, infrared (IR) spectroscopy, gas chromatography (GC), mass spectrometry (MS), high performance gel permeation chromatography (HPGPC), UV spectrum scanning and nuclear magnetic resonance (NMR). Homogeneity and MW are mostly measured by HPLC [44,45], HPGPC [45] and SEC [46,47] technologies. After being completely hydrolyzed by trifluoroacetic acid, the hydrolysate is separated and analyzed by HPLC, GC [48], or GC-MS [48]. Detection of functional groups is commonly carried out by IR or FT-IR. In order to determine the composition of the main chain and branched chain, methylation analysis combined with GC-MS is an effective method to determine the linkage types of glycosyl residues [22,49].

Nuclear magnetic resonance (NMR) spectra, including ^1^H, ^13^C were widely used to determine the abnormal structure, position and linkage sequence of glycosyl residues [27,50]. Circular dichroism (CD) spectra can directly analyze the conformational structure, usually by characterizing the Congo red polysaccharide complexes to determine the conformational characteristics of the solution at 540 nm by semi-quantitative estimation by UV [27,51]. Moreover, recent research shows that *Aloe vera* polysaccharide can be determined by the use of size exclusion chromatography (SEC)–multi-angle laser light scattering (MALS)–differential refractive index (DRI) [52].

In general, acemannan has molecular weights in the range of about 1000–1600 kDa [53,54]. Most plant polysaccharides consist of two or more kinds of monosaccharides, such as rhamnose (Rha), mannose (Man), fucose (Fuc), glucose (Glc), xylose (Xyl), arabinose (Ara) and galactose (Gal), while acemannan is mainly composed of mannose (84.9%), glucose (7.2%), and galactose (3.9%) [53]. Various polysaccharides, including acemannan, have been isolated from aloe [55,56]. Acemannan, which consists of β-(1,4)-linked mannose residues, is the most widely studied aloe polysaccharide, [57]. Acemannan is partially acetylated at the C-2 and C-3 positions that exhibit an acetyl:mannose monomer ratio of approximately 1:1, and also contains some side chains attached to C-6 like galactose [22,31,58]. Ulteriorly, linkages between monomers in acemannan were analysed by ^13^C-NMR, and the results indicated acemannan contains a single-chain backbone of β-(1,4) mannose with β-(1,4) glucose inserted into the backbone and α-(1,6) galactose branching from the backbone [54]. By using methylation, high performance size-exclusion chromatography coupled with multiangle laser light scattering (HPSEC-MALLS), NMR, GC-MS and scanning electron microscopy (SEM) techniques were used to analyse ASP. The research showed that ASP consists of β-(1,4)-glucomannans with acetyl groups, which may attach to the *O*-2, *O*-3 or *O*-6 positions of mannopyranosyl residues in the backbone as mono-, di-, or tri-acetylated forms [59] This suggests that ASP is probably acemannan. Acemannan was found to have a Man: Glc:Gal:GalA:Fuc:Ara:Xyl ratio of 120:9:6:3:2:2:1 with traces of Rha and GlcA. Relevant studies have provided direct evidence for the backbone of dextran, but also questioned the structure of the side chain. Further linkage analysis in acemannan treated with endo-(1→4)-β-D-mannanase yielded 4-Manp (53%), Manp-(1→ residues (26%), 2,4-Manp (3%), 3,4-Manp (1%), 4,6-Manp (1%), 4-Glcp (5%), 4-Xylp (1%), Xylp-(1→ residues (2%), Galp-(1→ residues (5%). The structure is further characterized by the method of NMR analysis, and the acemannan has a majority of a-Galp-(1→residues linked to *O*-2, *O*-3, or *O*-6 of →4)-β-Manp-(1→ residues, with ∽16→4)- β-Manp-(1→ residues between the side chains [60].

## 3. Biological Activities of Acemannan

### 3.1. Immunomodulation Activity

**Figure 2:**
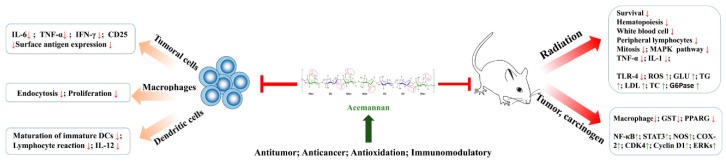
Pharmacological effects of acemannan in vitro and in vivo.

A large number of in vitro and in vivo studies have confirmed the immunomodulatory activity of acemannan on splenic lymphocyte, macrophage and dendritic cells. Acemannan, an important immunoenhancer, can enhance the lymphocyte response to alloantigen (Table 2, Table 3 and Figure 2). And the mechanism may be related to the release of IL-1 from the ordered nuclear cells under the protection of alloantigen [63,64]. The spleen, which combines the adaptive immune system and the innate immune system, is the largest secondary immune organ of the body [65,66]. Acemannan from *Aloe vera* can activate effectively regulate immunity. In [61], radiation-induced mortality of mice was significantly decreased when the mice were administered with acemannan at a dose of 150 mg/kg body weight by oral gavage for 7 days. The findings showed that the survival of mice treated with acemannan for 7-day pretreatment or post-treatment increased by 60 and 20%. Acemannan could the upregulate the cytokines like TNF-α and IL-1 and improve hematopoiesis, such as peripheral lymphocytes counts, spleen cellularity and spleen index. Similarly, hematopoiesis of C57 mice injected with 1 mg/mouse were obviously stimulated. What’s more, acemannan has a greater stimulatory activity for white blood cell (WBC) counts and spleen cellularity as well as on the absolute numbers of lymphocytes, neutrophils, monocytes in irradiation-induced myelosuppression mice [67]. Similarly, the mitogenic activities of splenocytes were obviously increased as splenic lymphocytes from spleen of Swiss albino mice were cultivated with the acemannan [53]. Consistent with previous studies, actanin can stimulate the antigenic and mitotic responses of human lymphocytes, but not mitosis itself [64].

Macrophages are an important part of the monocyte macrophage system and have a variety of functions in the immune response [68]. Highest enhancement in NO production or IL-1/IL-6 with acemannan through MAPK via binding with toll-like receptors, indicates the importance of the acetyl groups in macrophages from TBI mice [53]. Acemannan has a stimulating effect by enhancing the respiratory burst, phagocytosis, and killing of *Candida albicans* by murine peritoneal macrophages. 38% of *Candida albicans* died after 10 min of exposure to acemannan, compared with 0.5% in the control group. When mice peritoneal macrophages were incubated with acemannan for 60 min, 98% of the yeast was killed, compared with 0% in the control group [69]. Moreover, a mechanism of action for acemannan in activating RAW 264.7 cells has been proposed. In RAW 264.7 cells from a mouse macrophage cell line, acemannan stimulates the production of macrophage cytokines, nitric oxide release, surface molecule expression and cell morphology. Interleukin-6 (IL-6) and tumor necrosis factor-α (TNF-α) were produced in a dose-dependent manner, and morphological changes of the cells were observed. The increasing of surface antigen expression is stimulated by an increase in the mixture of interferon-γ (IFN-γ)-acemannan [70].

Dendritic cells (DCs) initiate and regulate highly pathogenic and specific adaptive immune responses, which are the core of the development of immune memory and tolerance [71]. Studies found that acemannan promoted nonspecific immunity, cellular immunity and humoral immunity. The immunomodulatory activities of acemannan were previously investigated in dendritic cells by culturing in a medium supplemented with GM-CSF, IL-4 and acemannan. Phenotypic analysis for the expression of class II MHC molecules and major co-stimulatory molecules such as CD40, CD54 B7-1 and B7-2 indicated that acemannan could significantly induce maturation of immature DCs though increasing allogeneic mixed lymphocyte reaction (MLR) and IL-12 production [42].

### 3.2. Anti-Cancer Activity

Colon cancer is one of the major causes of morbidity and mortality around the world. Inflammatory bowel disease is a chronic inflammatory disease, which can increase the risk of colorectal cancer (Table 2, Table 3 and Figure 2) [72,73]. Acemannan-induced macrophages increase cytotoxicity by increasing the production of nitric oxide in chicken bone marrow cell culture [74]. An experiment found that administration of PAG, rich in acemannan, significantly reduced the multiplicity of colonic adenomas and adenocarcinomas in colon cancer mice treated with an azoxymethane and dextran sodium sulfate. The study confirmed that PAG reduced the activation of nuclear factor kappa B (NF-κB) through the activation of peroxisome proliferator-activated receptor gamma, leading to the inhibition of inducible nitric oxidesynthase and cyclooxygenase-2 expression. What’ more, the expression and phosphorylation of signal transducer, activator of transcription 3 and cell cycle progression-inducing cellular factors including cyclin-dependent kinase 4, cyclin D1 and extracellular signal-regulated kinases 1/2 were decreased by PAG, resulting in inhibition of colorectal cancer [75]. Acemannan exhibited macrophage-activating activity in ICR mice implanted with sarcoma 180 cells compared with an untreated group. Results confirm that the macrophage-activating activity of acemannan shown in vitro is correlated with the antitumor activity in vivo [37].

Acemannan exhibited antiproliferative effect on murine (SpMC) and human cells (PBMC) and several tumoral cell lines of T lymphocytic origin though inhibiting the expression of activation markers (CD3–CD25 (+) cells). This also showed that acemannan has a dual effect on normal cells and tumor cells, inhibiting the activation of tumor cells and increasing the activity of normal cells [76]. Antigen expression and tumor killing ability of macrophage dysfunction. Therefore, macrophages are an excellent target for immunotherapy of tumors. Acemannan, as a biological response modifier and a potent murine B- and T-cell stimulator, could enhance the antigen recognition by inducing tumor cell cytotoxicity in murine peritoneal macrophage cells [77].

### 3.3. Antioxidant Activity

Lots of in vitro and in vivo experiments (Table 2 and Table 3) have indicated that acemannan has scavenging effects on free radicals [78,79] and ABTS [53]. The chelating activity and reduction ability of iron ion were verified. The cellular studies have showed that the polysaccharides could inhibit the production of reactive oxygen (ROS), thus reducing the damage caused by oxidative stress [80]. Numerous evidences insinuated that acemannan was capable of mitigating radiation-induced oxidative damages in vivo. It is well known that radiation can destroy the biological macromolecules by free radicals [61]. Study has proved that the multiple acetylated polysaccharides have antioxidation effect and can reduce the damage of DNA [81]. Study has proved acemannan reduces the radiation-induced oxidative by activating microphage via TLR-4 receptors [53]. Antioxidants protect biological systems from free radical-induced oxidative damage by scavenging or inhibiting free radical production [82]. The acetyl and hydroxyl groups of acemannan are respectively involved in free radical scavenging and formation [53].

### 3.4. Gastric and Intestinal Activity

Probiotics can promote the growth of *Lactobacillus* and *Bifidobacterium* species in the colon, resulting in the production of short chain fatty acids (SCFAs) by fermentation. Beneficial fermentation products can reduce the risk of non-communicable chronic diseases, including some types of cancer, such as colorectal cancer [89,90]. A recent study has confirmed that acemannan has advantages in inducing growth in bacteria such as *Bifidobacterium* and *Lactobacillus*. By co-culturing acemannan with *Bifidobacterium* and *Lactobacillus*, it was found that the number of beneficial colon bacteria increased significantly, followed by an increase in the synthesis of SCFAs, like acetate, propionate, and butyrate, found during fermentation of human feces with acemannan using qPCR [62]

### 3.5. Neuroprotective Activities

The neuroprotective effect of acemannan on humans has recently attracted considerable attention. Some non-starch polysaccharides, such as galactomannan and glucomannan extracted from bacteria and plants, have been shown to induce biological effects through direct or indirect mechanisms, including immune regulation of antioxidant and antidiabetic activities, as well as gastrointestinal and probiotics activities. Placebo-controlled experimental studies show that the consumption of glucomannans and galactomannans, acemannan improved cognitive performance in the middle age of mental fatigue [91]. The results provided that the improvement in memory performance following a mixture including acemannan was not related to changes in blood glucose. 

### 3.6. Hepatoprotective Effect

The antigenotoxic and chemopreventive effect of acemannan on (B[a]P)-DNA adducts was investigated in vitro and in vivo. Acemmannan could decrease [H-3]B[a]P-DNA adduct formation in primary rat hepatocytes and rat by increasing glutathione S-transferase activity [83].

## 4. Acemannan in Dentistry

**Figure 3:**
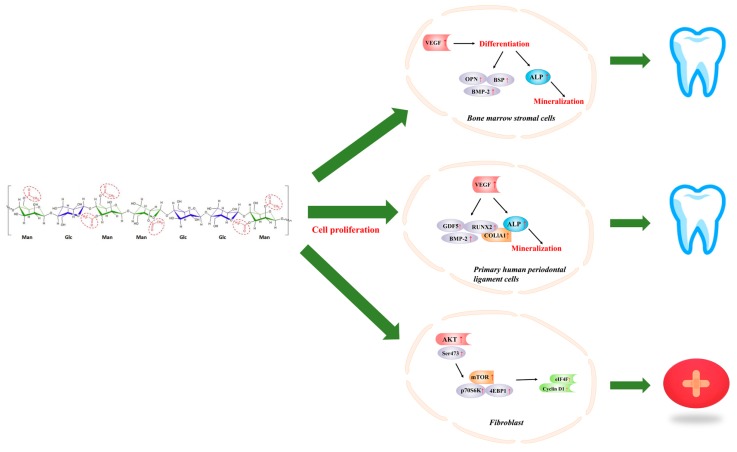
Application of acemannan in dentistry and wound healing.

Periodontal ligament cells are treated with plants to induce mineral deposition. Although there are few studies in this area, current evidence suggests that plants have a potential role in the treatment of periodontal disease [92]. Many research groups have demonstrated the role of acemannan in dentistry (Table 2, Table 3, Figure 3).

Numerous evidences insinuat that acemannan is capable of enhancing bone formation by stimulating primary rat bone marrow stromal cell (BMSC) proliferation, differentiation into osteoblasts, and extracellular matrix synthesis in vivo. New DNA synthesis, VEGF, BMP-2, alkaline phosphatase activity, bone sialoprotein, osteopontin expression, and mineralization was significantly improved in BMSCs treated with acemannan at a dose of 8 mg/mL. Moreover, acemannan-treated Sprague–Dawley rats in a tooth extraction model had higher bone mineral density and faster bone healing in vivo. The results proved acemannan could function as a bioactive molecule inducing bone formation [46].

Acemannan, as an active substance, increased mRNA expression and mineral deposition of BMP2. Results showed acemannan significantly increased periodontal ligament cell proliferation, type I collagen and alkaline phosphatase activity, upregulation of growth/differentiation factor 5, runt-related transcription factor 2, VEGF, BMP2 and mineral deposition in vitro. In addition, acemannan significantly accelerated cementum, new alveolar bone and periodontal ligament formation [84]. Another study has found acemanan also promoted mineralization of human dental pulp cells [85]. Those studies suggest acemannan may be a natural biomolecules that promotes the regeneration of periodontal tissues. Treatment of human papillary cells by acemannan can lead to proliferation of dental pulp cells, activation of alkaline phosphatase, type I collagen, BMP-2, BMP-4, vascular endothelial growth factor, dentin saliva protein expression and mineralization. It showed acemannan are biocompatible with the pulp [86]. 

**Figure 4:**
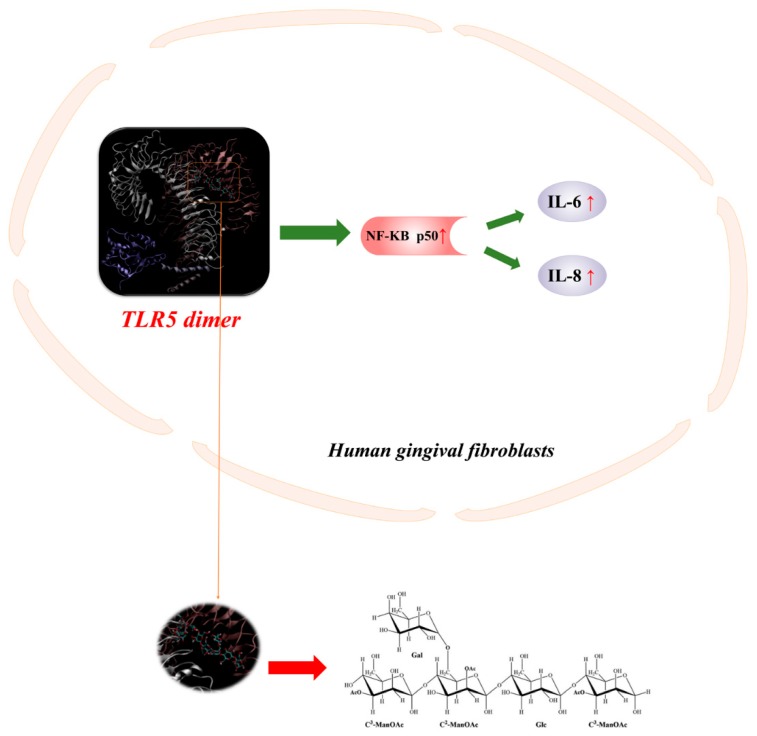
Acemannan induction of IL-6/-8 expression and p50/DNA binding in gingival fibroblasts via TLR5/ / NF-κB-dependent signaling pathway.

Computer simulation of the mechanism between polysaccharides and bacteria has been proved (Figure 4). The results indicated that acemannan stimulates IL-6/-8 expression at mRNA and protein levels and significantly increases p50/DNA binding. Computer simulations showed that the monomer/dimer single-chain acemannan molecule interacts with the TLR5 flagellin recognition site and has a high binding affinity. The first demonstration of acemannan induction of IL-6/-8 expression and p50/DNA binding in gingival fibroblasts via TLR5/ / NF-κB-dependent signaling pathway [33].

Further clinical random controlled trial has also shown that polysaccharides can promote the increase of dental bone density. After surgical removal of alveolar bone, 99 volunteers (18–24 years old) were randomly divided into an acemannan sponge group and control group for alveolar bone healing. Three months later, the mandibular partial bone impacted third molar was removed. Results showed the percentage and change rate of alveolar bone X-ray density in the acemannan treatment group were significantly higher than those in the control group at 3 months after operation, which proved that acemannan has the effect on increasing bone density and tooth socket healing in the third molar of the mandibular part [93]. Another clinical trial investigated 37 children aged 7–11 years with a diagnosis of reversible pulpitis. After the infected dentin was completely removed by surgery, the teeth exposed to the pulp tip were randomly divided into two treatment groups: acemannan or calcium hydroxide. Histopathological observation of the teeth showed that the histopathological response of the acemannan treatment group was significantly better than that of the calcium hydroxide treatment group, and the overall success rate of acemannan was 72.73% [19]. The results showed that the natural polysaccharide material is biocompatible and can promote the formation of dentin. In the future, it can be used as a direct pulping material for human deep deciduous teeth.

## 5. Acemannan in Wound Healing

Ulcers are one of the most common lesions in the mouth and can cause discomfort and pain, especially in patients with systemic diseases. Wound healing of oral ulcers is a complex process involving a large number of different cell types in migration, proliferation, differentiation, clearance of damaged tissue, and formation of extracellular matrices to protect the oral cavity [94,95]. Clinical wound healing tests conducted with acemannan showed that polysaccharides can accelerate healing time, reduce pain, and have no side effects (Table 2, Table 3, Figure 3) [96].

Acemannan was found to possess oral wound healing functions in human gingival fibroblasts and rats, including stimulating the expression of keratinocyte growth factor-1 (KGF-1), vascular endothelial growth factor (VEGF) and type I collagen production in cells at a dose of 16 mg/mL, and ameliorating oral wound healing in rats with the dose of 2% acemannan daily [7]. A skin patch clinical trial of 100 healthy subjects showed that 5% acemannan had a significant effect in reducing ulcer size and pain, and no subjects had an allergic reaction or side effects to acemannan. There was no significant difference in blood routine between 7 days before and after application of acemannan [16]. 

However, the role and potential molecular mechanisms of acemannan in skin wound healing are largely unclear. Therefore, two studies were conducted in vitro and in vivo. On one hand, the first study used mouse skin wound model and skin primary fibroblasts as experimental materials, and rapamycin and AKT inhibitor VIII were used to determine the key role of AKT/mTOR signaling pathway in promoting acemannan skin wound healing. It was found that acemannan can significantly accelerate wound closure and fibroblast proliferation in mice. The mechanism was that acemannan promoted the expression of cyclin D1 in cultured fibroblasts via AKT/mTOR signaling pathway and enhanced the activity of eukaryotic translation initiation factor-4f (eIF4F) and increased translation of cyclin D1, ultimately promoted wound healing in the skin [87]. On the other hand, further research for the effect of acamannan on the healing of rat skull defect was studied. The results showed that the bone surface and bone volume of the rats in the treatment group increased significantly. Histological observation showed that the bone matrix density of the rats in the polysaccharide treatment group was significantly higher than that of the control group. The results demonstrated that acemannan was an effective natural bioactive substance that promotes bone growth, increases bone surface, bone volume and bone density [88].

## 6. Structure-Activity Relationship

Numerous studies have shown that the structural characteristics of plant polysaccharides, such as molecular weight, chemical composition, branching structure and conformation, affect their biological activity [11,97,98]. First, acetyl is an important reactive group in acemannan. Studies have shown that different processing conditions will affect the acetylation of polysaccharides. FTIR analysis showed that the degree of acetylation of industrial freeze-dried samples was reduced by about 20%, while spray drying, refraction window drying and radiation zone drying were reduced by about 20%. Acetylation also further affected the physical properties of polysaccharides, and different drying methods also promoted critical behaviors of flow behavior, from shear thinning behavior [99].

Moreover, acetylation and deacetylation, adding and removing an acetyl group, is of great significance to the physical and biological activities of polysaccharides [80]. Studies have shown that acetylation of acemannan enhances its viscosity and thermal stability due to the stability of acetyl and hydroxyl groups [53]. On the acemannan structure, the acetyl group is the only non-glycan functional group. Thus, the acetyl group may be a functional domain of acemannan, which affects at least some of its topological structure, biological activity and physical properties. Deacetylation of acemannan, by treating with NaOH, changed the water solubility, three-dimensional structure, hydrophobicity and orderly packing of its molecules. Removing acemannan’s acetyl groups decreases its bioactivity by reducing its inductive activity on cell proliferation. Thus, acetyl groups play an important role in the structure and physical/biological properties of acemannan [14].

The degree of acetylation not only affects the physical properties of the polysaccharide, but also affects the biological activity of the polysaccharide. By comparing with other semi-emulsions, one study found that lower branches, shorter chains, and higher acetylation appeared to promote the immunostimulatory activity of these polysaccharides [49].

A recent mechanistic study also confirmed the conclusion that acemannan acetyl groups regulate the immune system, while hydroxyl groups participate in free radical scavenging. By binding MAPK to toll-like receptors, in the production of NO or the binding of IL-1/IL-6 to acemannan, acetyl has the strongest effect in TBI mouse macrophages, and the results of the study can also guide molecular modification [53].

## 7. Applications of Acemannan

Numerous studies have shown that *Aloe vera* gel can promote wound healing and reduce the damage caused by radiation to the skin. Thus, *Aloe vera* gel is made into various active materials, such as antibacterial non-woven fabric [100], polyelectrolytes, aloe polysaccharide/bacterial cellulose composite membrane [101], antibacterial and anti-oxidant edible films [102].

In fact, acemannan is the main active substance in the gel. One study tested the mechanical properties, thermal properties and antibacterial activity of hybrid gels made by mixing chitosan and acemannan (CS-AC). The results demonstrated that the CS-AC hybrid gel could provide promising future development leads and other biomaterial scaffolds for wound therapy [103].

Acemannan has also found many applications in the medical industry. An acemannan-rich sponge was more easily absorbed than a complex inorganic material, and can also act as a scaffold to provide migration and attachment of cell growth factors, because the sponge can not only effectively fix the blood clot in orbit. Acemannan has a significant effect on the healing of alveolar bone and further promotes bone formation. This means that acemannan may be a natural biopolysaccharide material for bone regeneration [46].

In addition, acemannan can be used as an effective vaccine adjuvant to prevent certain avian viral diseases. Because acemannan is able to effectively and permanently increase the activation capacity of chicken systemic immune chamber macrophages (especially macrophages from blood and spleen after intramuscular injection) by promoting the production of NO, ultimately regulating immunity [17,74,104]. Other studies have found that *Aloe vera*, rich in β-polysaccharides like acemannan, could be used as a prebiotic fermented milk, but the specific mechanism needs further study [105].

## 8. Conclusions and Future Prospects

*Aloe vera* is one of the most popular medicinal plants, widely used in the prevention or treatment of skin diseases, metabolic diseases, cardiovascular diseases and cancers throughout the world. Acemannan is one of the main components responsible for various biological activities, especially anti-tumor activity, and has received more and more scientific attention in recent years. Acemannan, isolated and purified from *Aloe vera*, has a variety of biological activities and is widely used in functional foods and pharmaceutical products. This paper mainly expounds the structural characteristics of acemannan, including the composition, molecular weight, configuration and position of sugar chains of monosaccharides. The biological activity and mechanism have been studied for many years. Acemannan has good anti-tumor effect, immune activity, antioxidant activity, the ability to promote wound healing and promote bone hyperplasia.

Although great improvement has been made these years, more efforts need to be done in this research area. For instance, among polysaccharide extraction techniques, such as water extraction and ethanol precipitation, the extraction rate of ethanol is relatively high, the operation is simple and environmentally friendly. However, these extraction methods still have the disadvantages of being time-consuming and high cost. What’s more, the degree of acetylation of the polysaccharide will be affected if the experimental conditions are not appropriate, like high temperature. Therefore, the extraction and separation of polysaccharides from *Aole vera* should be vigorously developed and utilized by adopting new, simple, efficient and cost-effective methods such as ionic liquid-water two-phase systems and nano-iron technology. Secondly, it is important to elucidate the chemical structure and chain conformation of polysaccharides to study their biological activities, so the precise structure of polysaccharides (higher order structure) and the relationship between structure and biological activity need further characterization and evaluation for their biological activity. Moreover, the application of acemannan in the medical field is of great significance and should be well established. On the other hand, in order to fully determine the effects of acemannan metabolites on human health due to the limitations of in vitro studies, it is necessary to further utilize powerful new technologies for human nutrition, clinical and epidemiological studies, such as “omics technology” (genomics, transcriptomics, metabolomics, and proteomics) and bioinformatics to elucidate the different mechanisms whereby acemannan effects its biological activities.

## Figures and Tables

**Table 1:** Summary of the extraction, purification, characterization methods and structural studies of acemannan.

Source	Extraction, Fractionation, Purification	Structural Characterization Method	MW (kDa)	Monosaccharide Composition	Structural Feature	Yield	Reference
Fresh gel	Water extraction (Homogenization, centrifugation mixed with 3 volumes of ethanol, lyophilization)	LC; FT-IR; SEC; ^1^H-NMR	190–220	Man: Glc: Gal = 57:22:17	*O*-(Acetyl-d-Manp)-*O*-(acetyl-d-Manp)-*O*-(d-Glap)-*O*-(acetyl-d-Man)	---	[14]
Fresh gel	Water extraction; separation (Shodex Sugar KS-804 column)	^13^C-NMR; SEM ^1^H-NMR; FT-IR	150-190	Man: Glc: Gal = 65:17:17	Single-branched galactose at C6 of the second acetylated mannose residue	0.04%	[33]
Frozen gel	Ultrafiltration cell membrane (Fractionated by ultrafiltration cell with MW cut-off membrane)	HPLC (BIOSEP SECH400 column); GC (SP2330 glass-capillary column); ^1^H-NMR; IR	>500	Man: Glc = 97:3	Galp-(1→residues link to *O*-2, *O*-3, or *O*-6 of →4)-β-Manp-(1→ residues, with ∽16→4)-β-Manp-(1→ residues between the side chains	2%	[42]
Fresh gel	Water extraction; separation (homogenization, centrifugation, alcohol precipitation, dialysis, lyophilization)	HPLC (Shodex Sugar KS-804 column); GC–MS; ^13^C-NMR	≥800	Man (77.18%); Glc (15.3%); Gal (4.9%); Ara (0.7%); Rha (0.2%); Fuc (0.34%); Xyl (0.7%)	β-(1→4)	0.2%	[46]
Fresh gel (1 year old)	Water extraction (Homogenization, centrifugation with 80% v/v alcohol, ammonium sulfate precipitation, lyophilization)	GC-MS; SEC; ^13^C-NMR	1100	Man: Glc = 15:1	*O*-2, *O*-3, and *O*-6 of→4)- β-Manp-(1→residues to single α-Galp-(1→side chains	---	[47]
Fresh gel	Water extraction; (Homogenization, centrifugation mixed with 3 volumes of ethanol, wash with ethanol, lyophilization)	HPGPC; FTIR; GLC-MS; TGA	1020	Mannose (84.9%): glucose (7.2%); galactose (3.9%)	(1→4)-Linked mannose/glucose 2,3,6-tri-*O*-acetyl-mannose,2,6-di-*O*-acetylglucose,6-acetyl-*O*-glucose, 3,6-di-*O*-acetyl-glucose	---	[53]
Frozen gel	Water extraction (Homogenization, centrifugation, alcohol precipitation, lyophilization)	GC–MS; Ion-chromatograph; ^13^C-NMR	1100	Man: Glc: Gal: GalA: Fuc: Ara: Xyl = 120:9:6:3:2:2:1	→4)-β-Manp-(1→ and →4)-β-Glcp-(1→ residues in 15:1 ratio	---	[60]
Fresh gel (3 years old))	Water extraction (Homogenization, centrifugation, supernatant mixed with 3 volumes of ethanol, pellet)	FACE; FT-IR; SEC	281	Man (62.9%); Glc (13.1%); Gal (0.6%)	---	1.7%	[61]
Frozen gel	Water extraction; (depigmentation, deproteinization)	CR; GC-MS; PACE	---	Man (86.87%); Glc (0.05%); Gal (12.68%); Ara (0.38%)	β-(1→4)	0.32%	[62]

Fluorophore-assisted carbohydrate electrophoresis (FACE); Congo red (CR); Liquid chromatography (LC); Size exclusion chromatography(SEC); Polysaccharide analysis by carbohydrate gel electrophoresis (PACE); Thermogravimetric analysis (TGA).

**Table 2:** Biological activity of acemannan in vitro.

Source	Target	Dose	Biological Activities	Action or Mechanism	Reference
Fresh gel	Human gingival fibroblasts	16 mg/mL	Oral wound healing	Proliferation (+); keratinocyte growth factor-1 (KGF-1) (+); VEGF (+); type I collagen production (+)	[7]
Fresh gel	Human gingival fibroblasts	10 mg/mL	Oral wound healing	IL-6 (+); IL−8 (+); p50/DNA (+); TLR5/NF-κB (+); Binds with TLR5 ectodomain flagellin recognition sites	[33]
Freeze-dried gel	Immature dendritic cells (mice)	100 µg/mL	Immunomodulatory	Induce maturation of immature DCs; mixed lymphocyte reaction; IL-12 (+)	[42]
Fresh gel	Bone marrow stromal cell (BMSC) (rat)	8 mg/mL	Periodontal tissue regeneration	BMSC proliferation (+); vascular endothelial growth factors (VEGF) (+); ALPase activity (+); bone morphogenic protein-2 (BMP-2) (+); bone sialoprotein (BSP) (+); osteopontin(OPN) (+); mineralization (+)	[46]
Fresh gel	RAW 264.7 cells (mouse)	100 µg/mL	Immunomodulatory	IL-6 (+); TNF-α (+); surface antigen expression (+); IFN-γ (+)	[70]
Fresh gel	Tumoral cells (murine, human)	0.6 mg/mL	Antitumor	Spontaneous proliferation (−); CD25 (+)	[76]
Fresh gel	Peritoneal macrophages (mice)	500 µg/mL	Induced tumor cell cytotoxicity	Endocytosis (+); murine macrophage stimulation	[77]
Fresh gel	*Lactobacillus*, *Bifidobacterium*, human fecal bacteria	3 g/L	Prebiotics	Growth (+); butyrate (+); propionate (+); SCFA (+)	[62]
Fresh gel	Hepatocytes (rat)	0.4–250 µg/mL	Antigenotoxic	3H]B[a]P-DNA adduct formation (−)	[83]
Fresh gel	Human periodontal ligament cells	4 mg/mL	Periodontal tissue regeneration	Cell proliferation (+); RUNX2 (+); GDF5(+); VEGF (+); BMP2 (+); COL1 (+); ALP (+); mineral deposition (+)	[84]
Fresh gel	Human periodontal ligament cells, pulpal cells	1 mg/mL	Periodontal regeneration	BMP2 mRNA (+) and protein (+)	[85]
Fresh gel	Human primary dental pulpal cells	---	Periodontal regeneration	Proliferation (+); alkaline phosphatase (+), type I collagen (+); BMP-2 (+); BMP-4 (+); vascular endothelial growth factor (+); dentin sialo protein expression (+); mineralization (+)	[86]
LGM Pharma	Skin primary fibroblasts (mice)	150 µg/mL	Cell proliferation	Cyclin D1 (+); eukaryotic translation initiation factor-4F (eIF4F) (+); activation of AKT/mTOR	[87]

**Table 3:** Biological activity of acemannan in vivo.

Source	Model	Target	Dose	Administraction	Biological Activities	Action or Mechanism	Reference
Fresh gel	---	Sprague Dawley rats (male)	2%	External application	Wound healing	Reduced oral wound areas	[7]
Dring gel (200:1)	Implanted with sarcoma 180 cell	ICR mice	1 mg/mouse	Injection	Antitumor	Exhibited macrophage-activating activity	[37]
Fresh gel	Tooth extraction model	Sprague–Dawley rats (male)	32 mg/kg	External application	Accelerating bone formation	Bone mineral density (+); tooth socket healing (+)	[46]
Fresh gel	Irradiation at 2.14 Gy/min	Swiss albino mice (male)	50 mg/kg	Oral gavage	Immunomodulation/radioprotection; antioxidation	Scavenge free radicals; survival (+); mitogenic activity (+); hematopoiesis (+); activation of MAPK	[53]
Fresh gel	Radiation-induced mortality	Swiss albino mice (male)	150 mg/kg	Oral gavage	Immunomodulatory	Survival (+); peripheral lymphocytes (+);TNF-α (+); IL-1 (+)	[61]
Fresh gel	Radiation-induced myelosuppression	C57BL/6 mice (female)	1 mg/mouse	Injection	Hematopoiesis	White blood cell (+); spleen cellularity (+); lymphocytes (+); neutrophils (+); monocytes	[67]
Fresh gel	[3H]B[a]P	ICR mice (male)	50 mg/mouse	Oral gavage	Antigenotoxic	Glutathione-s-transferase (+);[3H]B[a]P-DNA adduct formation (-)	[83]
Fresh gel	---	Mongrel Dogs	---	Oral gavage	Accelerating bone formation	Induced bone, cementum, and periodontal ligament formation	[84]
Fresh gel	Full-thickness skin excisional wound	BALB/c mice (male)	2 mg/kg	Injection	Wound healing	Accelerated skin wound closure	[87]
Fresh gel	---	SpragueeDawley rats (female)	8 mg/sponge	External application	Bone regeneration	Integrate new bone with the old bone	[88]
